# Identifying schizophrenia stigma on Twitter: a proof of principle model using service user supervised machine learning

**DOI:** 10.1038/s41537-021-00197-6

**Published:** 2022-02-07

**Authors:** Sagar Jilka, Clarissa Mary Odoi, Janet van Bilsen, Daniel Morris, Sinan Erturk, Nicholas Cummins, Matteo Cella, Til Wykes

**Affiliations:** 1 Institute of Psychiatry, Psychology & Neuroscience, King’s College London, London, UK; 2 South London and Maudsley NHS Foundation Trust, London, UK; 3 Division of Mental Health & Wellbeing, Warwick Medical School, University of Warwick, Coventry, UK

**Keywords:** Schizophrenia, Psychology

## Abstract

Stigma has negative effects on people with mental health problems by making them less likely to seek help. We develop a proof of principle service user supervised machine learning pipeline to identify stigmatising tweets reliably and understand the prevalence of public schizophrenia stigma on Twitter. A service user group advised on the machine learning model evaluation metric (fewest false negatives) and features for machine learning. We collected 13,313 public tweets on schizophrenia between January and May 2018. Two service user researchers manually identified stigma in 746 English tweets; 80% were used to train eight models, and 20% for testing. The two models with fewest false negatives were compared in two service user validation exercises, and the best model used to classify all extracted public English tweets. Tweets classed as stigmatising by service users were more negative in sentiment (*t* (744) = 12.02, *p* < 0.001 [95% CI: 0.196–0.273]). Our linear Support Vector Machine was the best performing model with fewest false negatives and higher service user validation. This model identified public stigma in 47% of English tweets (n5,676) which were more negative in sentiment (*t* (12,143) = 64.38, *p* < 0.001 [95% CI: 0.29–0.31]). Machine learning can identify stigmatising tweets at large scale, with service user involvement. Given the prevalence of stigma, there is an urgent need for education and online campaigns to reduce it. Machine learning can provide a real time metric on their success.

## Introduction

Mental Health is frequently discussed on Twitter, and some service users may find a sense of community and a safe space for expression, support, and self-management information to help them cope with their mental health problems^1^. But Twitter may be harmful through allowing the propagation of stigmatising attitudes and ideas, which can become part of the narrative around mental health conditions and those who suffer from them. Stigma has negative effects on people with mental health problems by making them less likely to seek help^2,3^. The first stage of combating stigmatising attitudes is reliable identification, but it is difficult to police harmful and stigmatising tweets given the high tweet volume. Machine learning techniques could automatically identify and potentially block them or allow the targeting of online anti-stigma campaigns^4^.

Machine learning models have used social media data, for example, to identify symptoms of depression using the sentiment in a user’s content^5^. But models can be bias from the way data are collected (ascertainment bias), or as consequences of conscious or unconscious biases in human decision-making in the data used to train the models^6^. Evaluation metrics (e.g., accuracy, false negatives) need to be acceptable to the community of users who will benefit from them. All these issues are important in classification and we have taken the view that the essential components for an acceptable model are: supervision of machine learning models to avoid bias, iterative modelling to identify the best performing model, and full involvement of the community who will use the technology to increase acceptability.

This is a proof of principle study to understand if ‘machine learning can use service user rated tweets to reliably automate the identification of new tweets as stigmatising’? We chose to investigate stigma associated with schizophrenia because it is highly stigmatised on Twitter compared to other mental health or neurological disorders^7^, and little is known about its prevalence on popular social media platforms^8^.

## Results

### Service user manual coding

There was good interrater reliability (kappa = 0.75) between the two service user researcher ratings, and the final verification of the tweet classifications resulted in 100% agreement with those ratings. There were 299 tweets judged as stigmatising (40%), and 447 (60%) that were non-stigmatising, and this dataset (*n* = 746) was used for machine learning.

### Feature analysis: Do stigmatising tweets differ from non-stigmatising tweets?

Service user coded stigmatising tweets had more negative sentiment scores (non-stigmatising mean = 0.037 (*SD* = 0.269); stigmatising mean = −0.198 (SD 0.247); *t* (744) = 12.02, *p* < 0.001 [95% CI: 0.196–0.273]). Stigmatising tweets were also more subjective in content (*t* (682.31) = −10.55, *p* < 0.001 [95% CI: −0.286 to −0.197]), contained fewer numeric characters (*t* (604.21) = 3.17, *p* = 0.02 [95% CI: 0.042–0.180]), fewer punctuations (*t* (706.82) = 5.22, *p* < 0.001 [95% CI: 1.038–2.289]), and were shorter on average than non-stigmatising tweets (*t* (739.09) = 9.581, *p* < 0.001 [95% CI: 0.998–1.512]). A full list of feature statistics is available in Supplementary Table 1.

### Machine learning: Which model best predicted false negatives?

The 80% of tweets used to train the models maintained the class distributions of the whole dataset; 60% were non-stigmatising (*n* = 357) and 40% were stigmatising tweets (*n* = 239) and this also applied to our 20% test model.

The AUC indicates that the random forest is better able to distinguish between stigmatising and non-stigmatising tweets than the SVM (94% vs 92%). However, the SVM with a linear kernel produced the fewest false negatives, which was preferred by service users, followed by the random forest model (*n* = 3 compared to *n* = 11). The SVM also produced one fewer false positive (*n* = 10 compared to *n* = 11). We also investigated accuracy (overall agreement with service user coding) and the SVM had slightly better accuracy than the random forest (91% vs 87%) (see Supplementary Fig. 1 and Supplementary Table 2).

When classifying stigma prevalence in the testing set, the random forest classified 39% of tweets as stigmatising and the SVM classified slightly more (45%).

### Blind validation

After removing tweets from the blind validation dataset, 922 remained. Two service user researchers rated 440 tweets, and another two rated 482 tweets, and then all 922 tweets were classified by both the SVM and random forest. See Supplementary Fig. 2 for a full flowchart of the tweets used for this.

#### The SVM and batch 1

There was fair to substantial agreement between service user researcher ratings and the SVM in batch 1 (*κ* = 0.652, 95% CI [0.585, 0.719], *p* < 0.001; *κ* = 0.631, 95% CI [0.560, 0.702], *p* < 0.001). The number of false negatives was 55, 30.

#### The SVM and batch 2

There was fair to moderate agreement between service user researcher ratings and the SVM in batch 2 *(κ* = 0.305, 95% CI [.217, .393], *p* < 0.001; *κ* = 0.486, 95% CI [.412, .560], *p* < 0.001). The number of false negatives was 96 and 99 respectively.

The SVM found stigma in 43% of the tweets. The same percentage was found by the independent coder.

#### The Random forest and batch 1

There was fair to substantial agreement between the service user researchers and the random forest model in batch 1 (*κ* = 0.595, 95% *CI* [0.524, 0.666], *p* < 0.001; *κ* = 0.621, 95% CI [0.548, 0.694], *p* < 0.001) but with more false negatives (77 and 45).

#### The Random forest and batch 2

There was fair to moderate agreement between the service user researchers and the random forest model in batch 2 (*κ* = 0.291, 95% CI [0.205, 0.377], *p* < 0.001; *κ* = 0.443, 95% CI [.369, .517], *p* < 0.001), but higher numbers of false negatives (105 and 112).

The random forest found stigma in 39% of tweets. The same percentage was found by the independent coder.

### Unblind validation

After removing tweets, 797 remained (see Supplementary Fig. 4).

#### SVM

There was substantial agreement between the service user researcher and SVM (*κ* = 0.667, 95% CI [0.616, 0.718], *p* < 0.001), with 102 false negatives. The SVM found stigma in 42% of tweets.

#### Random forest

There was substantial agreement between the service user researcher and the random forest model (*κ* = 0.614, 95% CI [0.561, 0.667], *p* < 0.001), but with 139 false negatives. The random forest found stigma in 36% of tweets.

In comparison to the prevalence of stigma found by these models, the researcher found stigma in 51% of tweets.

See Supplementary Tables 3–7 and Supplementary Figs. 3 and 5 for a detailed breakdown of validation scores.

### Big data analysis

The SVM with a linear kernel was the best performing model on our service user defined evaluation metric—false negatives and the validation measures. It was used to classify all tweets in our large corpus of English tweets (*n* = 12,145). We found that 46.7% of tweets (*n* = 5,676) were identified as stigmatising.

### SVM tweet classification

Tweets identified by the SVM as stigmatising were significantly more negative in sentiment (*t* (12,143) = 64.38, *p* < 0.001 [95% CI: 0.29–0.31]) and more subjective (*t* (12,143) = −58.37, *p* < 0.001 [95% CI: −0.32 to −0.30]). See Table 1 for means and standard deviations.

**Table 1:** Sentiment and subjectivity scores for tweets identified as either stigmatising or not stigmatising by the Support Vector Machine.

	SVM rating	*N*	Mean	Std. deviation
Sentiment	Non-stigmatising	6469	0.08	0.25
	Stigmatising	5676	−0.22	0.26
Subjectivity	Non-stigmatising	6469	0.35	0.31
	Stigmatising	5676	0.66	0.27

Excluding retweets (*n* = 6168 tweets) did not affect these results and the retweeted data set had the same pattern as the total dataset (see Supplementary Table 8).

### Location: where do stigmatising tweets originate from?

Some users did not provide location data (*n* = 2,624, 21.6%). There were countries that had large numbers of tweets (e.g., USA, *n* = 4,958) with high proportions of stigmatising tweets (*n* = 2,700, 47.6%), but there were also countries that had a large number of tweets but lower levels of stigma e.g., Canada produced 933 tweets (3rd highest) but only 3.3% were stigmatising, and the UK produced 1,357 tweets (2nd highest) but only 7.6% were stigmatising. The sentiment of stigmatising tweets from the USA was more negative than Canada and the UK (USA, mean = −0.11 ± 0.29; Canada, mean = 0.02 ± 0.31; UK, mean = 0.01 ± 0.28) (*F* (6100, 2) = 106.99, *p* < 0.001). See Supplementary Table 9 for further detail and Supplementary Fig. 6 for word clouds of most common words from each countries’ negative tweets.

## Discussion

We describe a supervised machine learning pipeline where service user views are at the core of the process. This proof of principle modelling began after discussions with a national young person’s mental health advisory group, all of whom had personally read and been affected by stigmatising content online. They advised reliable identification of stigmatising tweets (false negatives) and features of stigma found in tweets. This study also involved service user researchers who classified and validated stigma in tweets for training and testing the machine learning models. We demonstrate that schizophrenia stigma on Twitter can be reliably identified using supervised machine learning models when developed collaboratively with individuals with lived experience of using mental health services.

Our linear SVM was the best performing model based on its ability to identify false negatives—a service user requirement. The SVM has previously been used to classify stigma (alongside seven other themes) in tweets related to mental health campaigns^9^. In this study, a clinician and student classified their tweets to train their SVM and achieved a mean test accuracy of 64% on their test data. Our SVM accuracy was 91%, which highlights the importance of involving service users, not only so that a model reflects their values, but also to highlight features that may be important to them.

After applying our SVM to our 12,145 English tweets, we found almost half of public tweets related to schizophrenia were classed as stigmatising. This is striking considering previous work investigating stigma in Alzheimer’s disease found only 21% of 6,583 tweets to be stigmatising^8^, but perhaps not unexpected because we know that schizophrenia is highly stigmatised on Twitter compared to other mental health or neurological disorders^7^.

There are, however, methodological considerations important here. The SVM’s tendency to overclassify tweets as stigmatising is likely due to its high susceptibility to noise^10^. To make predictions, new tweets must have the same features as the training tweets. We generated features from the words in our relatively smaller training dataset (n596), so when predicting stigma on new tweets, there may be words in the new tweets which were not present in the larger testing dataset (n12145). This would make the model reliant on the fewer features that are only present in the training tweets, and therefore make broader generalisations^10^. This is an important consideration as it highlights the need for larger training datasets to ensure models don’t over (or under) estimate prevalence.

Our service users wanted the accurate identification of stigmatising tweets to be the priority and agreed that false negatives (i.e., model predicts no stigma when it was stigmatising) be the primary evaluation metric. We found that our random forest was better able to distinguish between stigmatising and non-stigmatising tweets (higher AUC score) than our SVM. However, the purpose of involving service users is to ensure machine learning technologies are appropriate and useful for them from the very beginning. We selected the SVM because service users felt more strongly about not identifying stigmatising tweets correctly rather than losing positive tweets. To compare models further, we embedded blind and unblind validation tests that replicate our manual coding steps. Here, the random forest produced more false negatives in both validation stages compared to SVM. The random forest tends to overclassify tweets as non-stigmatising, whereas the SVM tends to overclassify tweets as stigmatising. Despite both these biases leading to *error*, when using these models to identify (and potentially remove) stigmatising tweets, errors on the SVM’s part result in the model being ‘too careful’. Service users stated that they would prefer this to the alternative of the model being ‘too lenient’ as in the case of the random forest.

The application of machine learning to mental health has previously demonstrated a range of benefits across the areas of diagnosis, treatment and support, research, and clinical administration^11^. Here, we propose ways to implement machine learning to understand conversations around stigma using social media. Previous work assessing stigma has suffered from low response rates, a reliance on surveys, and traditional media anchoring effects^12–15^. Applying machine learning models to social media data can offer huge benefits in combatting stigma and raising awareness of mental health problems^1^, which is important given the high prevalence of stigma found in our study.

We also highlight how stigma is propagated in different countries, and through retweets. Machine learning can be used to analyse real world big datasets effectively and efficiently, to create and evaluate tailored campaigns to fight stigma as it changes with time.

There is a complex relationship between bias occurring in the machine learning pipeline leading to a model, and the eventual bias of the model^16^. The development of our machine learning pipeline was carried out with and by service users and service user researchers, and in line with CPEDS guidance. This approach is one step towards minimising potential bias. There was good agreement by service users on what constitutes stigma, however, some tweets could be interpreted as stigmatising by one person, but not by another and this ambiguity is important to factor in when developing future models. The difficulty in developing a machine learning model in mental health reflects the heterogeneity in mental health conditions combined with societal and cultural factors which shape how individuals communicate and understand their mental health^17^. We propose that future work ensures that a more diverse group and a greater number of service users classify tweets, and that clinical, social, and cultural data are used to understand some of their personal reactions. This might then allow a more personalised approach to classification.

Our machine learning models were trained only on English tweets from all countries and did identify stigma, even where English is not the first language. However, stigma may exist in native languages with cultural nuances. Understanding stigma in the cultural context would inform future anti-stigma campaigns so they can be targeted more effectively.

We may have missed some tweets referring to schizophrenia. Comparisons between the Streaming API and the “firehose” suggest that the Streaming API may not sufficiently represent Twitter activity^18^. However, we did capture a broad sample of tweets using multiple time points. We also applied our SVM to all English tweets, irrespective of whether they were used in the machine learning or validation. This allowed us to understand the prevalence of public stigma on Twitter as a proxy of public attitudes. Future work should embed an iterative machine learning pipeline, to train and test models on new and more data, and re-test on data that reflects society beyond the time of data collection.

Mental health stigma needs to be monitored online as it can become part of the narrative around mental health conditions. We know that stigma has negative effects on people with mental health problems by making them less likely to seek help^2,3^, but our proof of principle study shows that we can reliably analyse large datasets with machine learning. There is still a need to minimise bias and involve the beneficiaries of future machine learning applications, in this case, mental health service users. Given the prevalence of schizophrenia-related stigma on Twitter, there is an urgent need for education and online campaigns to reduce stigmatising content, and machine learning models can be used to identify their effects.

## Methods

**Fig. 1:**
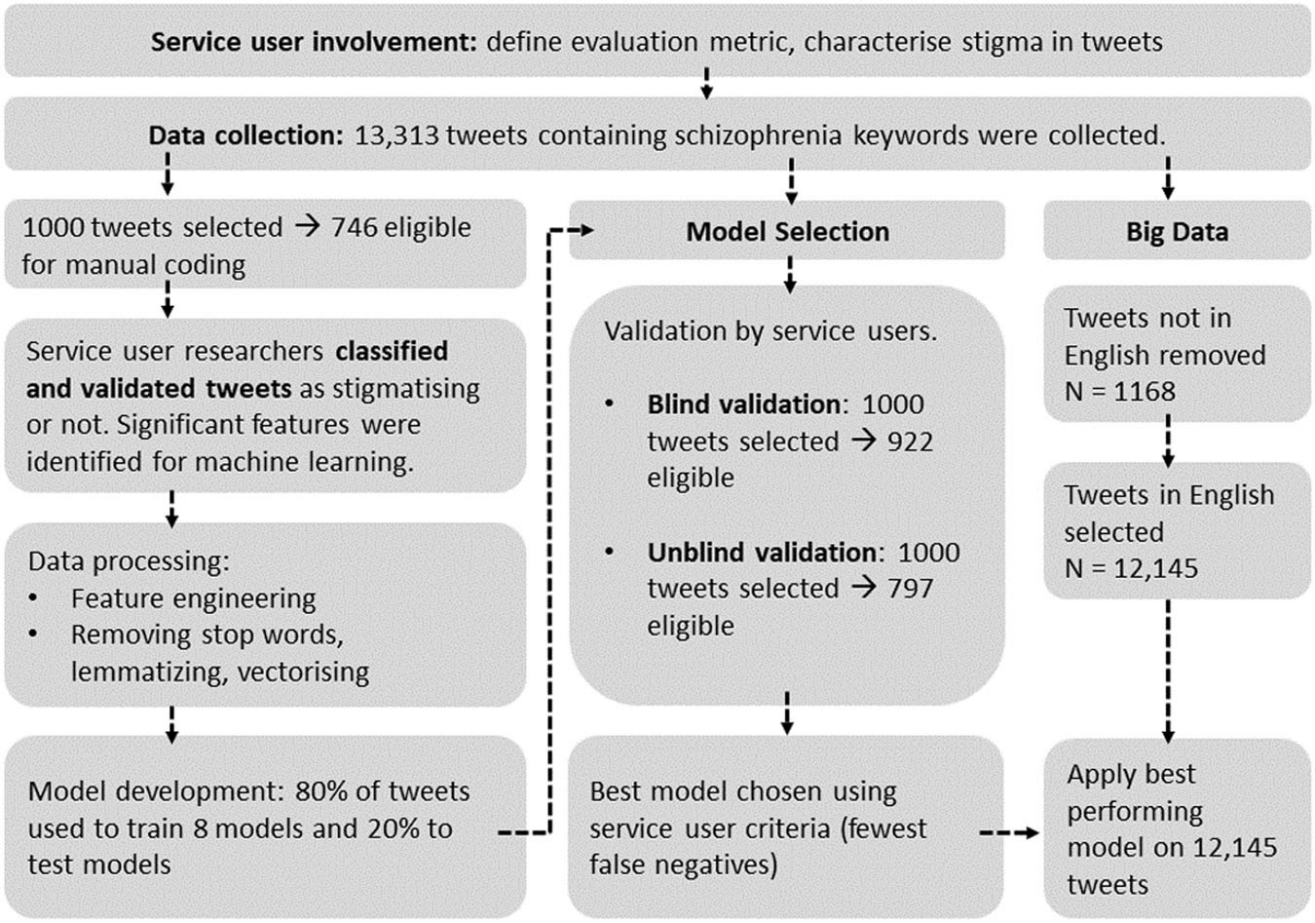
An overview of our methodology and data collection. An outline of the number of tweets used for each section of the methodology and how they were used.

We followed the Community Principles on Ethical Data Practices^19^ (CPEDP) to implement a machine learning pipeline to predict schizophrenia stigma in tweets. Our pipeline involved (a) setting evaluation metrics and characterising tweets with service users (b) collecting relevant tweets; (c) service user ratings of tweets; (d) processing tweets (including featuring engineering, and vectorising); (e) developing and validating machine learning models with service users; (f) applying the best performing model to new tweets to understand the prevalence of schizophrenia stigma. Figure 1 outlines these steps. Ethics approval was not required as we used publicly available, non-sensitive data, that was anonymized.

### Ethical approach

CPEDP is a crowdsourced ethics code for data sharing across the science community. The principles focus on sourcing, sharing, and implementing data that causes no harm and maximises positive impact. We ensured that the beneficiary—people with lived experience of mental health problems—were involved from the study conception and curating the datasets, as well as refining and validating the machine learning models. Data collection took place securely and through Twitter’s official Streaming Application Programming Interface (API) and were stored anonymously on university-owned encrypted servers and all analyses took place on these servers.

The CPEDP is an initiative started by a group of data scientists who wanted a data science version of the ‘Hippocratic Oath’. The inception of this was at an event on 6th February 2018 in San Francisco (https://dataforgoodexchangesf.splashthat.com/). The principles are now highlighted here (https://datapractices.org/manifesto/) and the lead author (Sagar Jilka) was one of the early signatories of this manifesto (signature number 1819). The principles state that as data teams, we aim to:

1. Use data to improve life for our users, customers, organisations, and communities.
2. Create reproducible and extensible work.
3. Build teams with diverse ideas, backgrounds, and strengths.
4. Prioritise the continuous collection and availability of discussions and metadata.
5. Clearly identify the questions and objectives that drive each project and use to guide both planning and refinement.
6. Be open to changing our methods and conclusions in response to new knowledge.
7. Recognise and mitigate bias in ourselves and in the data we use.
8. Present our work in ways that empower others to make better-informed decisions.
9. Consider carefully the ethical implications of choices we make when using data, and the impacts of our work on individuals and society.
10. Respect and invite fair criticism while promoting the identification and open discussion of errors, risks, and unintended consequences of our work.
11. Protect the privacy and security of individuals represented in our data.
12. Help others to understand the most useful and appropriate applications of data to solve real-world problems.

We applied these principles to our work by addressing and adopting several points on this manifesto. These include:

- Point 1: We used our data to identify schizophrenia stigma on Twitter in accordance with concerns that service user’s had about this stigma.
- Point 3: People with lived experience of mental health services designed the study, collected, and analysed the data. Our team was mixed in terms of gender and ethnicity.
- Point 5: Working with service users, we had a clear goal of identifying stigma towards schizophrenia on social media with machine learning models. Addressing this goal throughout the study naturally structured our research around a key plan.
- Point 7: By having service users form the categories for our machine learning algorithm, we removed any of our own bias as researchers from the process.
- Point 9: We agreed as a group not to publish the text within tweets, as users can be easily identified if the tweet is searched in Google.
- Point 11: While we gathered a wide range of data from the Tweets we obtained, including user names and locations, we ensured that no identifiable information was represented in our research and removed any unnecessary identifying data as soon as possible within the data we worked with.
- Point 12: Within our paper, we have highlighted key applications of our research such as the potential to utilise machine learning to detect stigma as an alternative to administering surveys and as a way to monitor the effectiveness of awareness campaigns, allowing for the creation and evaluation of tailored campaigns”.

### Service user involvement

We consulted a national young person’s mental health advisory group (YPMHAG, https://www.kcl.ac.uk/research/ypmhag) all of whom who had previous experience of using mental health services and had personally read negative and stigmatising tweets. They provided advice throughout the project. They advised that model success should be based on predicting the fewest false negatives (FN); where a tweet is stigmatising, but the model classifies it as not stigmatising (i.e., type II error). Members also suggested features which may help to identify stigmatising tweets.

### Data collection

Public tweets were extracted between January and May 2018 in real time for a period totalling 48 h during these times:

Round 1 (30/01/18); 24 h (preliminary round)

Round 2 (15/03/18); 6 h

Round 3 (17/04/18 & 18/04/18); 6 h

Round 4 (24/04/18 & 25/04/18); 6 h

Round 5 (10/05/18 & 11/05/18); 6 h

We used Twitter’s API^20^ via the Tweepy Python library^21^ to collect tweets. Tweepy is secure and requires a Twitter-approved application through the official Twitter developer’s webpage.

Tweets were captured if they contained keywords taken from a previous study on mental health stigma^7^: ‘schizophrenia’ or ‘schizophrenic’ or ‘psychosis’ or ‘psychotic’ or ‘schizo’. The extraction process captured all tweets with any occurrence of these keywords, anywhere in the tweet.

Five rounds totalling 48 h of data collection were completed, over two days to ensure that tweets were extracted across the whole day. The public tweets relating to Schizophrenia (*n* = 13,313) formed three datasets; for machine learning, validation, and our big data analysis on stigma prevalence.

### Service user manual coding for supervised machine learning

Two hundred tweets from each of the five extraction rounds were randomly selected using Python’s rand function (total = 1000) and after removing non-English tweets left 746 tweets that formed the machine learning dataset. Two service user researchers independently coded these tweets as stigmatising or not, and their inter-rater reliability assessed. Some tweets were excluded because there was no context (e.g., “*??????????psychotic??????*”).

We involved additional independent service user researchers to validate the classifications through two steps:

1. Two service users coded the tweets as stigmatising or not and if they disagreed on the classifications, a third service user researcher resolved the coding to produce the final classification.
2. To verify the final classifications of the tweets, a fourth service user researcher coded a random 20% sample.

### Feature engineering

Feature engineering is the application of domain knowledge to potential model inputs, with the goal of creating a feature set that is optimised to predict stigma^22^. A list of features is provided below. We extracted features based on previous twitter work and features that were identified by our service user advisory group are indicated with a *

1. *Sentiment:* Sentiment analysis has been used to extract people’s opinions on healthcare-related topics^23^. Sentiment identifies positive, neutral, or negative text and we considered this to be an important feature in stigma detection (scores closer to −1 are very negative, +1 are very positive and 0 is neutral). Sentiment for each tweet was analysed using a python library called TextBlob. Words with a stronger degree of sentiment will have a greater influence on the score and we considered this to be an important feature in stigma detection.
2. *Subjectivity:* Subjectivity refers to personal opinion, emotion or judgement, and stigmatising tweets might be more subjective (scores closer to +1 and factual information scores closer to 0). Each tweet was analysed to score the subjectivity of its content again using TextBlob. We wanted to investigate whether stigmatising tweets were more subjective in nature than non-stigmatising tweets.
3. *Length of tweet:* Shorter tweets are also associated with stronger opinions^24^, and we test whether, stigmatising tweets, which are based more in opinion than fact, are shorter than non-stigmatising tweets.
4. Punctuation*: Each tweet was analysed for the proportion of punctuation it contained to investigate whether stigmatising tweets contained more punctuations (e.g., exclamations marks) than non-stigmatising tweets. The improper use of grammar has been associated with stigma in tweets^8^ so we tested suggested how the proportion of punctuation in tweets is associated with stigma.
5. The number of uppercase words*: The YPMHAG noted that anger or rage is quite often expressed by writing in uppercase and we investigated whether stigmatising tweets were characterised by this feature.
6. *The average word length of a tweet:* This was computed by taking the sum of the length of all the words in a tweet and dividing it by the total length of the tweet. The average word length is an indicator of readability^25^ and we test whether stigmatising tweets are more or less readable based on their average word length.
7. *The number of words in a tweet:* Using fewer words is associated with a ‘clear communication goal’ which indicates negative emotion rather than objectivity^26^. We tested if stigmatising tweets used fewer words than non-stigmatising tweets.
8. *The number of characters in a tweet:* Twitter had increased its character limit to prevent people from ‘cramming their thoughts’^27^. It is therefore thought that as a user approaches the character limit (an increase in number of characters), they are having to put more thought into what they say, to ensure it is captured within the limit. We test whether stigmatising tweets are not well thought out as they may contain fewer characters.
9. The number of hashtags in a tweet*: YPMHAG members indicated that they would come across negative content while using hashtag search options.
10. The number of numeric characters in a tweet*: Members of the YPMHAG hypothesised that stigmatising content may contain words where a letter is replaced by its numerical form (e.g., the letter ‘A’ replaced with the number ‘4’).

### Data Analysis of service user manual coded tweets

The selection of relevant features, and the elimination of irrelevant ones, is still one of the central problems in machine learning^28^. Removing inappropriate features will minimise noise when fitting models^29^. We tested all features against the service user manually coded tweets to investigate whether our engineered features differed between the stigmatising and non-stigmatising tweets using independent sample, two-sided t-tests. Only significant features were then entered into the models.

### Data pre-processing for machine learning

Natural language processing methods converted the tweets into their numerical form which included removing stop words and lemmatising to remove noise^30^. These are described below:

### Dimensionality reduction

*Noise removal* The tweets were pre-processed to remove noise before vectorising^29^. This involved:

1. *Removing punctuation:* Punctuations were removed from the tweets.
2. *Tokenization*: Each tweet was separated into a list of individual words (i.e., tokens);
3. *Removing stop words*: Common, highly frequent words (e.g., *the, and, or*) were removed from the dataset;
4. *Lemmatising:* Each token was converted into its root form by removing inflectional endings (e.g., “typing” or “typed” into “type”). Lemmatising transforms words into ones that are less readable, but closer to their base meaning, and thus more suitable for comparison across tweets. Lemmatising also reduces the number of features in the dataset and therefore decreases the noise in the model.

Each tweet was vectorized using Term Frequency-Inverse Document Frequency^31^ (TF-IDF). This method of vectorizing is based on the weighting of words within the tweet, with the importance of the word within a tweet encapsulated by its weighting. This is considered along with the frequency of the word across all the tweets, where rarer words are given higher values (see equation below).

This weighting encapsulates the amount of information inherent in a word, based on a linguistic observation. For example, a noun or a verb may represent greater meaning but occur less often when compared to function words. In this variant, a weighting based on the document frequency (i.e., the number of tweets containing the word) is multiplied by the frequency of the word in the tweet, as outlined by Eq. (1).

1 $$\begin{array}{lll}w_{i,j} &=& tf_{i,j} \times \log \left( {\frac{N}{{df_i}}} \right)\\ tf_{i,j} &=& {{{\mathrm{number}}}}\,{{{\mathrm{of}}}}\,{{{\mathrm{occurrences}}}}\,{{{\mathrm{of}}}}\,i\,{{{\mathrm{in}}}}\,j\\ df_i &=& {{{\mathrm{number}}}}\,{{{\mathrm{of}}}}\,{{{\mathrm{documents}}}}\,{{{\mathrm{containing}}}}\,i\\N &=& {{{\mathrm{total}}}}\,{{{\mathrm{number}}}}\,{{{\mathrm{of}}}}\,{{{\mathrm{documents}}}}\end{array}$$

The formula to calculate the Term Frequency-Inverse Document Frequency of a text dataset.

The aim of this was to create feature vectors, where the machine learning algorithm learns to correlate the frequency of certain features (words) in a tweet with the stigma classification ratings given to a tweet. In this way, the supervised machine learning model is used to create the automatic classifier, which is learning to predict stigma classification of a new tweet based on the human-coded data it is trained on. The resulting classifier is then used to assign class labels to testing instances (i.e., new tweets) where values of the predictor features are known (i.e., the new tweet itself) but the value of the class label is unknown (i.e., the stigma level of the unrated tweet).

### Machine learning

In supervised machine learning, tweet data with known classifications (stigmatising or not) are used to train a model to predict the classification of new tweets. Given the novelty of this approach, we compared the ability of eight models previously used in health data^32^ to test their ability to predict stigma. These were: Random Forest^33^, Random Forest with Gradient Boost^34^, K-nearest neighbour^35,36^, Naive Bayesian Classifier^37^, Support Vector Machine (SVM), and SVM with three different kernels; linear, sigmoid, and poly^38–40^. These are described in detail below:

*Random forest* is a ‘tree‐based’ algorithm where multiple decision trees are built using a random assortment of features that are used to predict an outcome data label (i.e., stigma). Using a ‘majority vote’ system, the multiple decision trees in the random forest model predict a new sample (i.e., tweet), and the ultimate classification of this new sample (i.e., stigmatising or not) is based on the classification predicted by the majority of the decision trees.

*Gradient boosting* is a machine learning approach for classification problems where ‘tree models’ are composed of thousands of relatively simple decision trees. These models are trained iteratively by combining individual decision trees to optimise a specified evaluation metric. At each iteration, an additional decision tree is added to the “ensemble” of previously trained decision trees. Each new decision tree considers errors made in the previous iterations. In this way, the model “learns” its own shortcomings and introduces a new decision tree to address them.

*The K‐nearest neighbour* algorithm is one of the simplest machine learning models. The principle of K‐nearest neighbour is to find a predefined number of training data features (known as K) with known labels closest to the new data point and subsequently predict the new label based on the K‐nearest training points^41^. K‐nearest neighbour is a non‐generalising machine‐learning method as it simply ‘remembers’ all of the training data and selects the data labels closest to the new point. Despite its simplicity, K‐nearest neighbour is useful in a large number of classification problems and is often successful in classification situations where the decision boundaries are irregular. Because of this, K‐nearest neighbour is highly sensitive to the local data environments compared with the overall dataset.

*Support vector machines (SVM)* have strong theoretical foundations and excellent empirical successes. Given a training dataset of feature-label pairs, the SVM maps the training vectors into a higher dimensional space and finds a linear separating hyperplane with the maximal margin in this higher dimensional space^39,42^. SVM has a kernel function and new kernels are being proposed by researchers, including linear, polynomial, and sigmoid^43^.

*Naïve Bayes* is a classification technique based on Bayes’ Theorem with an assumption of independence among predictors^44^. A naive Bayes classifier assumes that the presence of a particular feature in a class is unrelated to the presence of any other feature. For example, a fruit may be considered to be an apple if it is red, round, and about 3 inches in diameter. Even if these features depend on each other or upon the existence of the other features, all of these properties independently contribute to the probability that this fruit is an apple, which is why it is known as ‘Naive’.

Our machine learning algorithms were trained and tested using Scikit‐learn version 0.17.1 in the Python programming language^45,46^. Independent models were also run by an independent coder to ensure machine model validity.

### Further evaluation metrics

Following the YPMHAG discussions, we report model accuracy, the area under the receiver‐operating characteristic curve metric (AUC), and the number of false positives, all common metrics in machine learning^47^. Accuracy answers the question ‘Overall, how often is the model correct?’ (determined by summing the number of true positives and true negatives and dividing by the total number of responses), while AUC measures how well a model can differentiate between two classes; a score of 1 indicates perfect differentiation, and a score of 0 indicates no ability to differentiate. When the AUC is 0.5, it means a model has the ability to separate classes.

As we aim to distinguish between tweets that are stigmatising or not, we also report AUC score and accuracy because false positives (i.e., non-stigmatising tweets that are identified as stigmatising) were deemed more acceptable by the YPMHAG than false negatives (i.e., stigmatising tweets that are not identified).

### Train/test split

There are no standardised ratios for separating data into training and test sets, however, the 80/20 split is commonly used in machine learning^41^ so this was adopted. We used a grid search method to find the best performing parameters for the models. This included:

*Random forest and gradient boost*: the number of trees in the forest were 10, 50, 100, 150, and 200 and the maximum depth of each tree was 10, 20, 30, 50, and none.

*K nearest neighbours:* we iterated through a range of *k* = 1 through to 25.

#### SVM

We tested multiple kernels based on scikit-learns hyperparameter tuning tutorial (https://scikit-learn.org/stable/auto_examples/model_selection/plot_grid_search_digits.html); kernel coefficient (gamma) for ‘poly’ and ‘sigmoid’ were 0.001 and 0.0001; and for all three SVMs (linear, poly, and sigmoid), the regularisation parameter (C) tested were 1, 10, 100 and 1000.

#### Naive Bayesian classifier

Naïve Bayes model parameter various smoothing (var smoothing) were tested using a range from 1 to 0.000000001.

### Bootstrapping/cross validation

To evaluate and overcome issues of overfitting, we carried out bootstrapping/cross-validation^22^. No model modifications were made during each run of the bootstrapping.

### Model validation

To ensure that the model performs as service users would expect, we carried out a further replication of both the modelling and manual coding on the top two models based on false negatives, AUC score, and accuracy. A further 2000 English tweets were extracted from our corpus of 13,313 tweets and compared the service user scores with the model predictions using the kappa statistic for interrater reliability in SPSS version 25^48^, as well as the number of false negatives identified by service user researchers.

We carried out two validation analyses:

- *Blind validation:* 1,000 unique tweets were split into two 500 tweet batches and given to four independent service user researchers. So that the classifications were manageable, two researchers were assigned to each batch to classify as stigmatising or not. Our top two models then classified the 1000 tweets before calculating the kappa statistic and number of false negatives. This allowed us to compare the performance of both models against the ratings of all the service user researchers.
- *Unblind validation:* A second batch of 1,000 unique English tweets were extracted and we used our two top models to classify them as stigmatising or not. Another service user researcher scored whether they agreed with each model’s rating, before calculating the kappa statistic and number of false negatives for each model.

### Big data analysis

We removed non-English tweets from our large corpus of 13,313 tweets and applied our best performing model (fewest false negatives and good validation) in a big data analysis to classify stigma.

We used all the Twitter data including retweets to measure the prevalence of schizophrenia stigma as it would appear for a user, but also investigated the effect of retweets. We used independent samples, two-sided t-tests to investigate if tweets identified by our model as stigmatising were more (1) negative in sentiment, and (2) more subjective, compared to non-stigmatising tweets, and if there were differences in our other engineered features. We also explored the proportions of stigmatising tweets by country and investigated differences in sentiment using ANOVA.

### Reporting summary

Further information on research design is available in the Nature Research Reporting Summary linked to this article.

## Supplementary information

Supplementary Material

Reporting Summary

## Data Availability

The datasets generated and analysed during the current study are available from the corresponding author on reasonable request.

## Code availability

The code relating to this work is available at the corresponding authors GitHub repository. The link to which is available upon request.
