# Supplementary material for: Identifying schizophrenia stigma on Twitter: a proof of principle model using service user supervised machine learning
Source: Schizophrenia (Heidelb). 2022 Feb 7;8(1):1. doi: 10.1038/s41537-021-00197-6 (PMC8821670; doi:10.1038/s41537-021-00197-6)
Supplement: Supplementary file 1 — Supplementary Material [file 41537_2021_197_MOESM1_ESM.pdf]

Supplementary Material

Feature Analysis

Supplementary Table 1. T-test results of engineered features. * indicates significant difference.

Features	**Non-stigmatising Tweets** Mean (SD)	**Stigmatising Tweets** Mean (SD)	*t*	*P*
Sentiment	0.04 (0.27)	-0.20 (0.25)	12.02	0.00*
Subjectivity	0.40 (0.33)	0.65 (0.29)	-10.55	0.00*
Average word length	6.96 (2.21)	5.71 (1.36)	9.58	0.00*
Length of Tweet	174.95 (251.44)	155.26 (200.55)	1.13	0.26
Number of characters	202.02 (292.64)	182.35 (229.29)	0.98	0.33
Number of hashtags	0.69 (1.84)	0.44 (4.54)	1.02	0.31
Number of numeric characters	0.17 (0.68)	0.06 (0.25)	3.17	0.01*
Punctuation	7.68 (4.70)	6.02 (3.95)	5.22	0.00*
Number of uppercase words	1.01 (2.66)	1.18 (3.78)	-0.72	0.47
Word count	28.07 (42.11)	28.09 (29.43)	-0.01	1.00

Machine Learning

Alongside the service user preferred metric (false negatives), we also highlight accuracy and Area Under the Curve (AUC) metric.

Supplementary Table 2. False negatives (n), accuracy and AUC score for each model tested, sorted by the service user criterion of fewest false negatives

Model	False Negatives (n)	False Positives (n)	Accuracy	AUC Score
**SVM Linear Kernel**	**3**	**10**	**0.91**	**0.92**
**Random Forest (holdout)**	**11**	**11**	**0.87**	**0.94**
Gradient Boost (holdout)	12	11	0.86	0.91
Naïve Bayes	17	25	0.72	0.72
K Nearest Neighbours (n4)	23	32	0.63	0.63
SVM Sigmoid Kernel	43	27	0.53	0.50
SVM Linear (Cross Validation)	57	56	0.85	0.50
SVM (Default Kernel; Radial Basis Function)	60	0	0.60	0.50
SVM (Poly Kernel)	60	0	0.60	0.50
Random Forest (Cross Validation)	72	49	0.84	0.82
Naïve Bayes (Cross Validation)	75	150	0.70	0.50
Gradient Boost (Cross Validation)	82	72	0.79	0.78
K Nearest Neighbours (n4) (Cross Validation)	146	149	0.60	0.59
SVM Sigmoid Kernel (Cross Validation)	284	26	0.58	0.50
SVM (Default Kernel = Radial Basis Function) (Cross Validation)	297	9	0.59	0.50
SVM (Poly Kernel) (Cross Validation)	298	8	0.59	0.50

Bootstrapping/cross-validation of the data didn't improve the model performance. The SVM with a linear kernel produced 57 false negatives, with an accuracy of 85% and AUC of 50%. The random forest produced 72 false negatives, with 84% accuracy and 82% AUC.

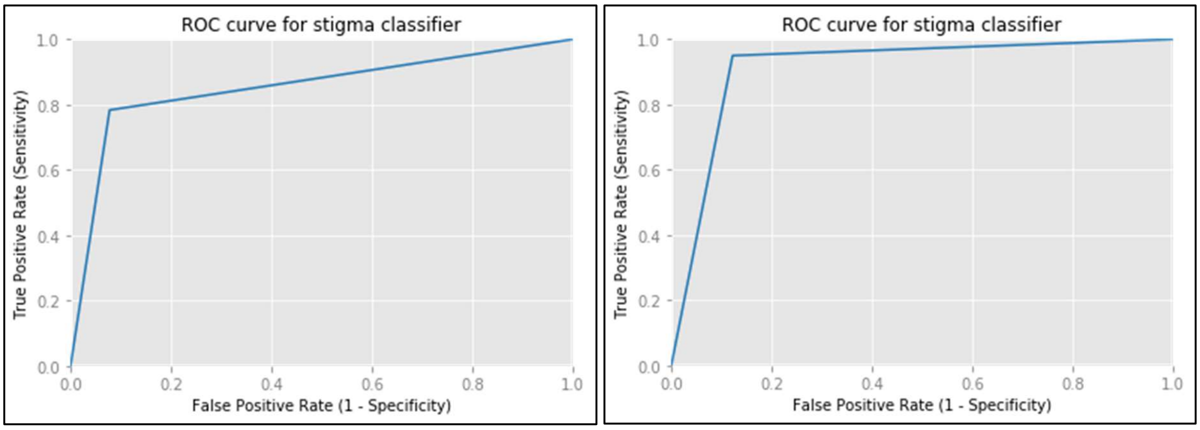

Supplementary Figure 1. The receiver operating characteristic curves for (left) the Random Forest model (AUC = 0.94), and (right) the SVM with linear kernel (AUC = 0.92).

Validation: Blind

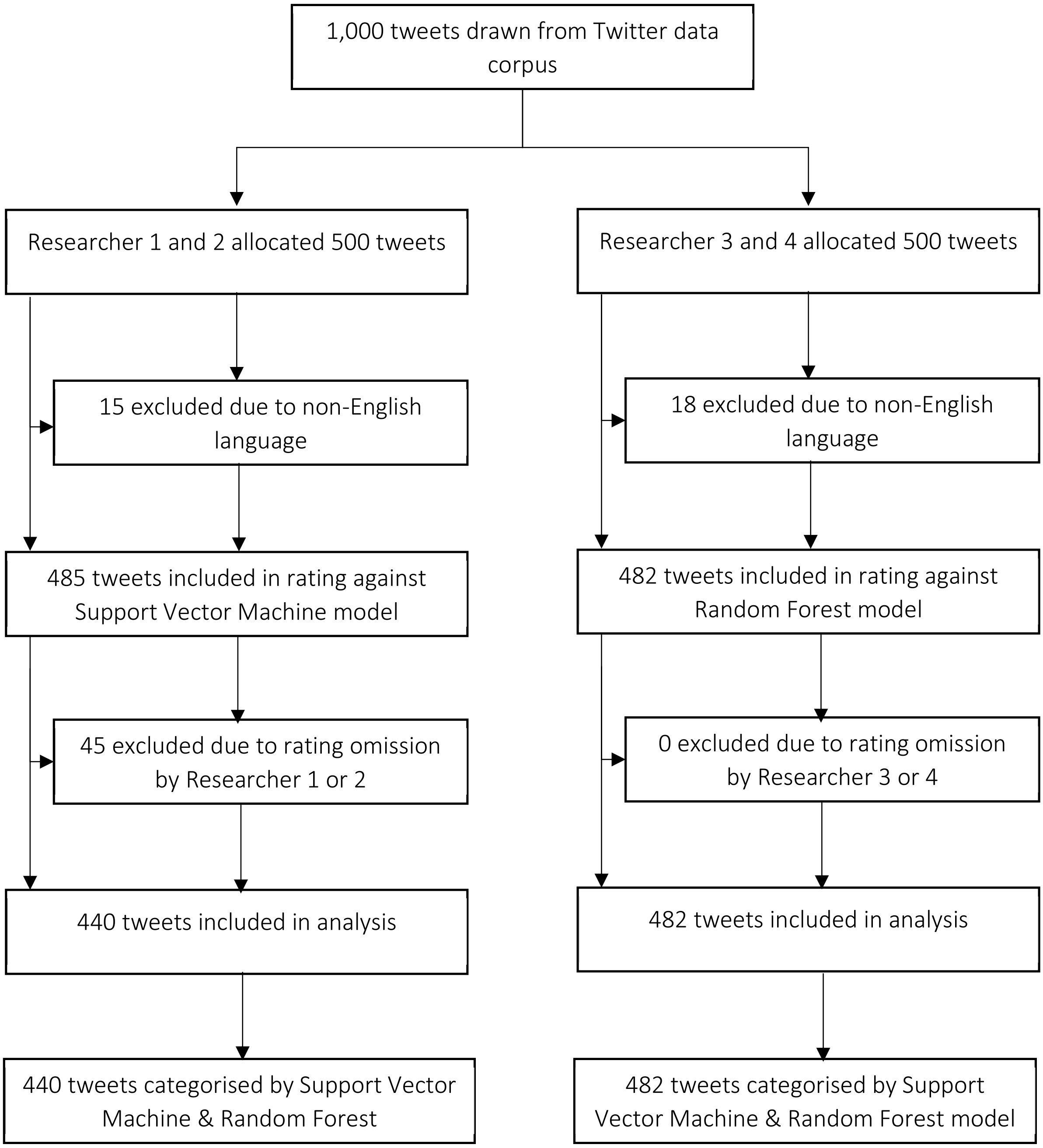

Supplementary Figure 2. Flowchart displaying tweets included at each stage of the blind validation

Service user researcher 1:

Between SVM and Researcher 1 (supplementary table 3): There was **fair** agreement between Researcher 1 and the SVM, κ = 0.305, 95% CI [0.217, 0.393], $p < .001$. Of the 440 tweets categorised, Researcher 1 and the model disagreed on 154 (35%). Of these, **96** of these were false negatives - instances where the model categorised the tweet as non-stigmatising but Researcher 1 categorised the tweet as stigmatising; 58 of these were false positives – instances where the model categorised the tweet as stigmatising but Researcher 1 categorised the tweet as non-stigmatising. Of the 440 tweets categorised, Researcher 1 and the model agreed on 286 (65%) of these (141 were categorised as stigmatising and 145 were categorised as non-stigmatising).

Between Random Forest and Researcher 1 (supplementary table 3): There was **fair** agreement between Researcher 1 and the Random Forest, κ = 0.291, 95% CI [0.205, 0.377], $p < .001$. Of the 440 tweets categorised, Researcher 1 and the model disagreed on 158 (36%). Of these, **105** of these were false negatives - instances where the model categorised the tweet as non-stigmatising but Researcher 1 categorised the tweet as stigmatising; 53 of these were false positives – instances where the model categorised the tweet as stigmatising but Researcher 1 categorised the tweet as non-stigmatising. Of the 440 tweets categorised, Researcher 1 and the model agreed on 282 (64%) of these (132 were categorised as stigmatising and 150 were categorised as non-stigmatising).

Supplementary Table 3. Confusion matrix displaying agreement between Researcher 1 and SVM & Random Forest models

Researcher 1 rating	SVM model rating			Random Forest rating		
	**Stigmatising n (%)**	**Non-stigmatising n (%)**	**Total n (%)**	**Stigmatising n (%)**	**Non-stigmatising n (%)**	**Total n (%)**
**Stigmatising n (%)**	141 (32)	**96 (22)**	237 (5)	132 (30)	**105 (24)**	237 (54)
**Non-stigmatising n (%)**	58 (13)	145 (33)	203 (46)	53 (12)	150 (34)	203 (46)
**Total n (%)**	199 (45)	241 (55)	440 (100)	185 (42)	255 (58)	440 (100)

Service user researcher 2:

Between SVM and Researcher 2 (supplementary table 4): There was **moderate** agreement between Researcher 2 and the SVM, κ = .486, 95% CI [.411, .560], $p < .001$. Of the 440 tweets categorised, Researcher 2 and the model agreed on 324 (73%) of these (182 were categorised as stigmatising and 142 were categorised as non-stigmatising). Of the 440 tweets categorised, Researcher 2 and the model disagreed on 116 (27%) of these (**99** of these were false negatives, 17 of these were false positives).

Between Random Forest and Researcher 2 (supplementary table 4): There was **moderate** agreement between Researcher 2 and the random forest model, κ = .0.443, 95% CI [.369, .517], $p < .001$. Of the 440 tweets categorised, Researcher 2 and the model agreed on 312 (71%) of these (169 were categorised as stigmatising and 143 were categorised as non-stigmatising). Of the 440 tweets categorised, Researcher 2 and the model disagreed on 128 (29%) of these (**112** of these were false negatives, 16 of these were false positives).

Supplementary Table 4. Confusion matrix displaying percentage agreement between Researcher 2 and SVM & random forest

Researcher 2 rating	SVM model rating			Random Forest rating		
	**Stigmatising n (%)**	**Non-stigmatising n (%)**	**Total n (%)**	**Stigmatising n (%)**	**Non-stigmatising n (%)**	**Total n (%)**
**Stigmatising n (%)**	182 (41)	**99 (23)**	281 (64)	169 (38)	**112 (25)**	281 (63)
**Non-stigmatising n (%)**	17 (4)	142 (32)	159 (36)	16 (4)	143 (33)	159 (36)
**Total n (%)**	199 (45)	241 (55)	440 (100)	185 (42)	255 (58)	440 (100)

Service user researcher 3:

Between Random Forest and Researcher 3 (supplementary table 5): There was **moderate** agreement between Researcher 3 and the Random Forest model, κ = .595, 95% CI [.524, .666], p < .001. Of the 482 tweets categorised, Researcher 3 and the model agreed on 386 (80%) of these (151 were categorised as stigmatising and 235 were categorised as non-stigmatising). Of the 482 tweets categorised, Researcher 3 and the model disagreed on 96 (20%) of these (**77** of these were false negatives, 19 of these were false positives).

Between SVM and Researcher 3 (supplementary table 5): There was **substantial** agreement between Researcher 3 and the SVM, κ = .652, 95% CI [.585, .719], p < .001. Of the 482 tweets categorised, Researcher 3 and the model agreed on 399 (83%) of these (173 were categorised as stigmatising and 226 were categorised as non-stigmatising). Of the 482 tweets categorised, Researcher 3 and the model disagreed on 83 (17%) of these (**55** of these were false negatives, 28 of these were false positives).

Supplementary Table 5. Confusion matrix displaying percentage agreement between Researchers 3 and the Random Forest

Researcher 3 rating	SVM model rating			Random Forest rating		
	**Stigmatising n (%)**	**Non-stigmatising n (%)**	**Total n (%)**	**Stigmatising n (%)**	**Non-stigmatising n (%)**	**Total n (%)**
**Stigmatising n (%)**	173 (36)	**55 (11)**	228 (47)	151 (31)	77 (16)	228 (47)
**Non-stigmatising n (%)**	28 (6)	226 (47)	254 (53)	19 (4)	235 (49)	254 (53)
**Total n (%)**	201 (42)	281 (58)	482 (100)	170 (35)	312 (65)	482 (100)

Service user researcher 4:

Between random forest and Researcher 4 (supplementary table 6): There was **substantial** agreement between researcher 4 and the Random Forest model, κ = .621, 95% CI [.548, .694], p < .001. Of the

482 tweets categorised, Researcher 4 and the model agreed on 398 (83%) of these (131 were categorised as stigmatising and 267 were categorised as non-stigmatising). Of the 482 tweets categorised, Researcher 4 and the model disagreed on 78 (17%) of these (**45** of these were false negatives, 39 of these were false positives).

Between SVM and Researcher 4 (supplementary table 6): There was **substantial** agreement between researcher 4 and the SVM, κ = .631, 95% CI [.560, .702], p < .001. Of the 482 tweets categorised, Researcher 4 and the model agreed on 397 (82%) of these (146 were categorised as stigmatising and 251 were categorised as non-stigmatising). Of the 482 tweets categorised, Researcher 4 and the model disagreed on 85 (18%) of these (**30** of these were false negatives, 55 of these were false positives)

Supplementary Table 6. Confusion matrix displaying agreement between Researcher 4 and random forest and SVM

Researcher 4 rating	SVM model rating			Random Forest rating		
	Stigmatising n (%)	Non-stigmatising n (%)	Total n (%)	Stigmatising n (%)	Non-stigmatising n (%)	Total n (%)
**Stigmatising n (%)**	146 (30)	**30 (6)**	176 (37)	131 (27)	**45** (9)	176 (37)
**Non-stigmatising n (%)**	55 (12)	251 (52)	306 (63)	39 (8)	267 (56)	306 (64)
**Total n (%)**	201 (42)	281 (58)	482 (100)	170 (35)	312 (65)	482 (100)

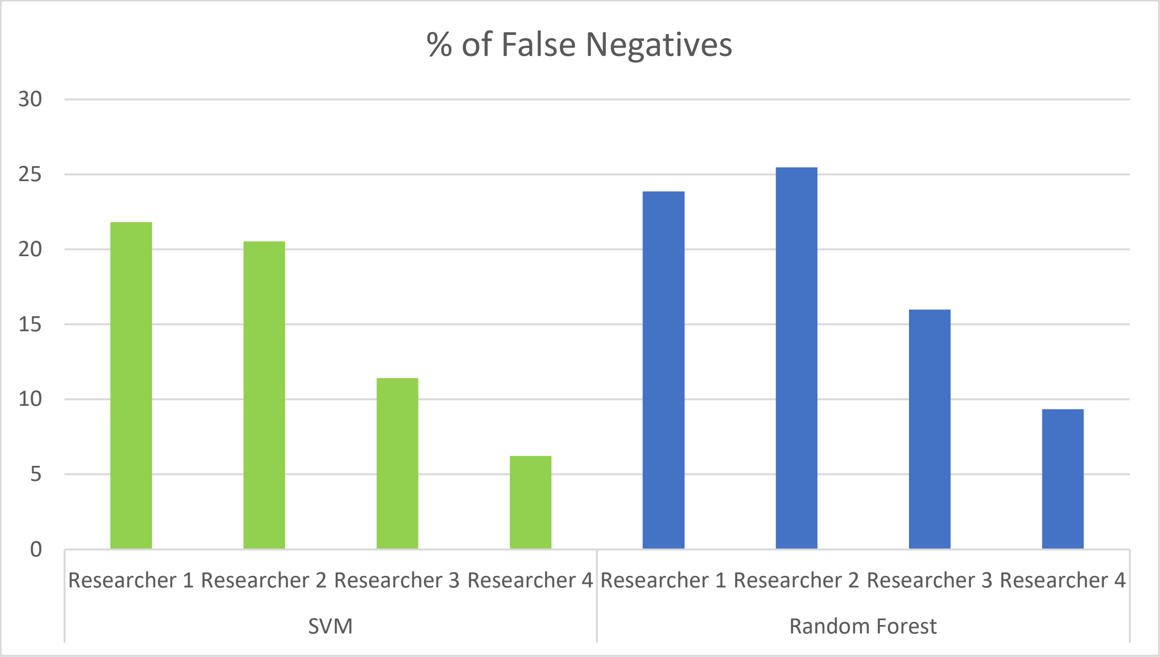

Supplementary Figure 3. Percentage of false negative classifications made by the machine learning models (SVM in green, and Random Forest in blue) when the raters were blind to model tweet rating

Validation: Unblind

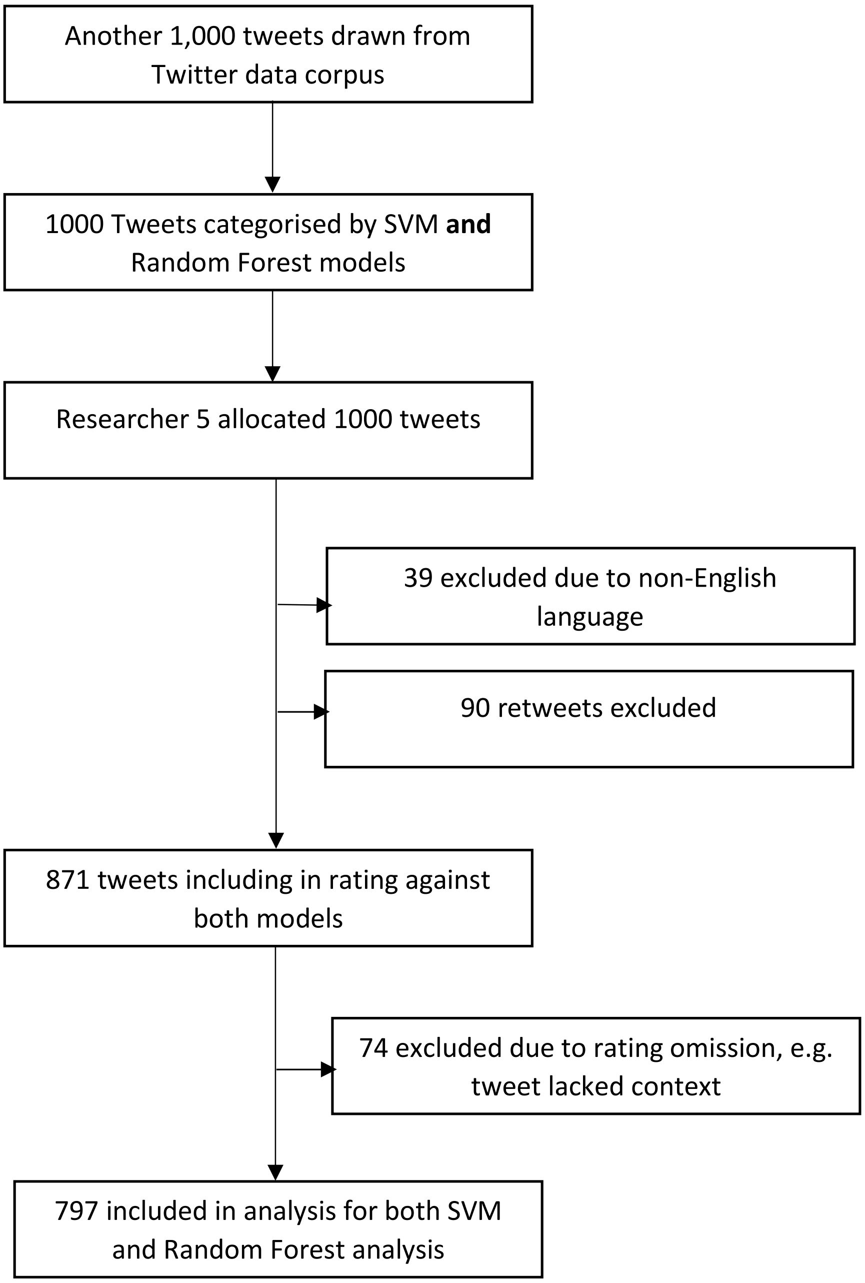

Supplementary Figure 4. Flowchart displaying tweets included at each stage of the unblind validation

Service user researcher 5:

Between SVM and Researcher 5: There was **substantial** agreement between Researcher 5 and the SVM, κ = .667, 95% CI [.616, .718], $p < .001$. Of the 797 tweets categorised, Researcher 5 and the model agreed on 664 (83%) of these (306were categorised as stigmatising and 358 were categorised as non-stigmatising). Of the 797 tweets categorised, Researcher 5 and the model disagreed on 133 (17%) of these (**102** of these were false negatives; 31 of these were false positives).

Between Random Forest and Researcher 5: There was **substantial** agreement between Researcher 5 and the random forest model, κ = .614, 95% CI [.561, .667], $p < .001$. Of the 797 tweets categorised, Researcher 5 and the model agreed on 642 (81%) of these (269 were categorised as stigmatising and 373 were categorised as non-stigmatising). Of the 797 tweets categorised, Researcher 5 and the model disagreed on 155 (19%) of these (**139** of these were false negatives, 16 of these were false positives).

Supplementary Table 7. Confusion matrix agreement between researcher 5 and the SVM and Random Forest.

**Researcher 5 rating**	**SVM model rating**			**Random Forest rating**		
	**Stigmatising n, (%)**	**Non-stigmatising n, (%)**	**Total n, (%)**	**Stigmatising n, (%)**	**Non-stigmatising n, (%)**	**Total n, (%)**
**Stigmatising n, (%)**	306 (38)	**102 (13)**	408 (51)	269 (34)	**139 (17)**	408 (51)
**Non-stigmatising n, (%)**	31 (4)	358 (45)	389 (49)	16 (2)	373 (47)	389 (49)
**Total n, (%)**	337 (42)	460 (58)	797 (100)	285 (36)	512 (64)	797 (100)

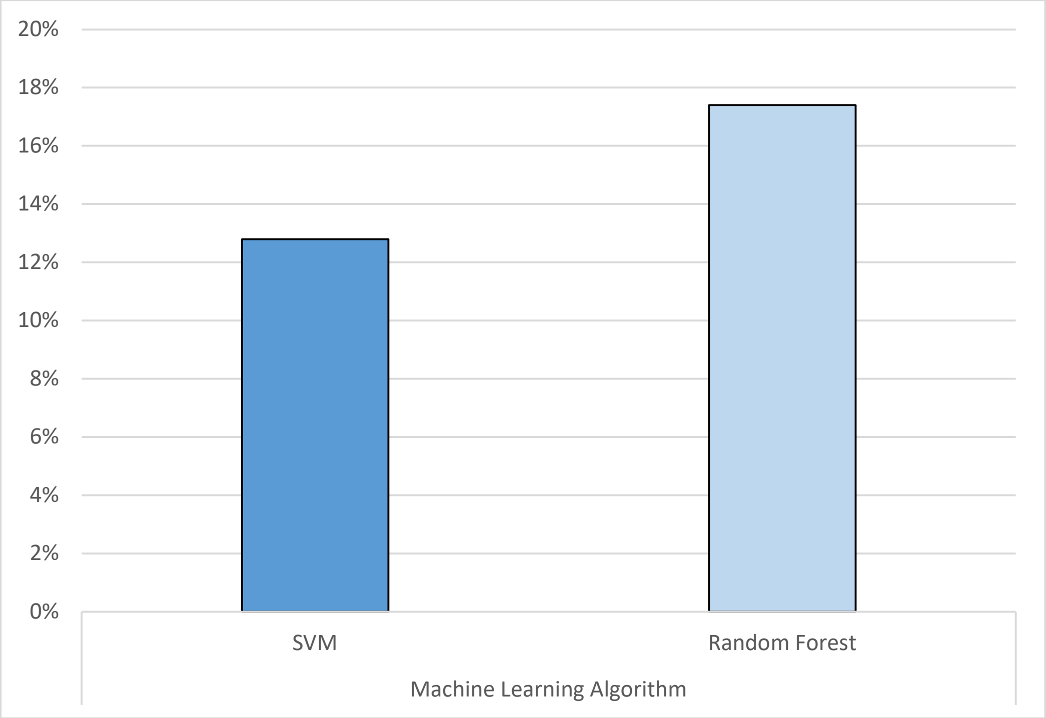

Supplementary Figure 5. Percentage of false negative classifications made by the machine learning models. Rater unblind to model tweet rating.

Big Data Analysis

Stigmatising vs non stigmatising tweets

After removing retweets and non-English tweets, tweets identified by the SVM as stigmatising were significantly more negative in sentiment (t (6,166) = 45.05., p < 0.001 [95% CI: 0.28 – 0.31]) and more subjective (t(6,166) = -43.18, p < 0.001 [95% CI:-0.33 - -0.30]). See table 10 for means and standard deviations.

Supplementary Table 8. Comparison of sentiment and subjectivity scores after removing non-English tweets, and retweets (n = 6,168 tweets).

	SVM Rating	N	Mean	Std. Deviation
Sentiment	Non-stigmatising	3338	0.06	0.26
	Stigmatising	2830	-0.23	0.25
Subjectivity	Non-stigmatising	3338	0.39	0.31
	Stigmatising	2830	0.70	0.26

Location: which countries did stigmatising tweets originate from?

Supplementary Table 9. Countries where tweets (>50) originated and their proportion of stigmatising tweets as a percentage.

**Country of Tweet**	**Total tweets (n)**	**Stigmatising tweets (n)**	**% of stigmatising tweets**
USA	4958	2700	47.6
United Kingdom	1357	433	7.6
Canada	933	187	3.3
**Country of Tweet**	**Sum tweets (n)**	**Sum Stigmatising tweets (n)**	**% range of stigmatising tweets**
Other countries*	1145	464	0.4 – 1.5

* countries included where total tweets > 50. These are: Australia, India, France, Germany, Ireland, The Netherlands, South Africa, Ecuador, Spain, Kenya, Pakistan.

Sentiment analysis between three countries with most tweets

Post-hoc tests were done with the one-way ANOVA to test variance in sentiment of stigmatising tweets between countries.

There was no significant difference between Canada and the United Kingdom (Mean difference = 0.02 (95% CI -0.02 – 0.05), p = 0.562.) but tweets were significantly more negative in the USA than Canada (Mean difference = 0.13 (95% CI 0.1 – 0.16), $p < 0.001$.) and the United Kingdom (Mean difference = 0.12 (95% CI 0.09 – 0.14), $p < 0.001$.)

Word clouds for stigmatising tweets from the three countries with the most tweets

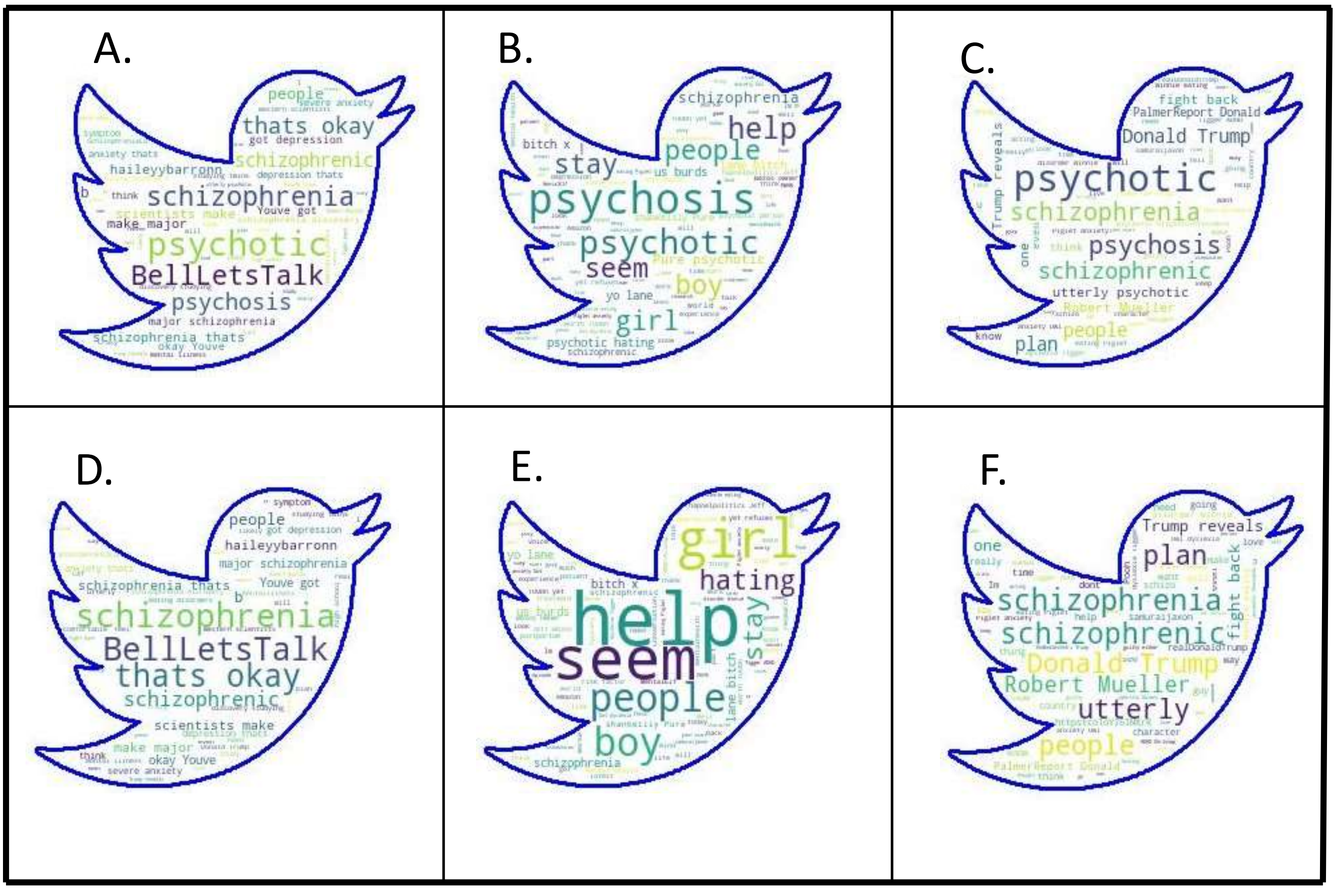

Supplementary Figure 6. Word clouds of the most common words in each country. A (Canada), B (United Kingdom) and C (USA) are all the full word clouds. D (Canada), E (United Kingdom) and F (USA) are the word clouds after removing the words 'psychosis' and 'psychotic' which were most common across all three. This makes the differences between countries clearer.

- END -
