# Supplementary material for: Identifying schizophrenia stigma on Twitter: a proof of principle model using service user supervised machine learning
Source: Schizophrenia (Heidelb). 2022 Feb 7;8(1):1. doi: 10.1038/s41537-021-00197-6 (PMC8821670; doi:10.1038/s41537-021-00197-6)
Supplement: Supplementary file 2 — Reporting Summary [file 41537_2021_197_MOESM2_ESM.pdf]

nature portfolio

Corresponding author(s): Sagar Jilka

Last updated by author(s): Nov 23, 2021

Reporting Summary

Nature Portfolio wishes to improve the reproducibility of the work that we publish. This form provides structure for consistency and transparency in reporting. For further information on Nature Portfolio policies, see our Editorial Policies and the Editorial Policy Checklist.

Statistics

For all statistical analyses, confirm that the following items are present in the figure legend, table legend, main text, or Methods section.

n/a	Confirmed	
☐	☒	The exact sample size (*n*) for each experimental group/condition, given as a discrete number and unit of measurement
☐	☒	A statement on whether measurements were taken from distinct samples or whether the same sample was measured repeatedly
☐	☒	The statistical test(s) used AND whether they are one- or two-sided<br>*Only common tests should be described solely by name; describe more complex techniques in the Methods section.*
☒	☐	A description of all covariates tested
☒	☐	A description of any assumptions or corrections, such as tests of normality and adjustment for multiple comparisons
☐	☒	A full description of the statistical parameters including central tendency (e.g. means) or other basic estimates (e.g. regression coefficient) AND variation (e.g. standard deviation) or associated estimates of uncertainty (e.g. confidence intervals)
☐	☒	For null hypothesis testing, the test statistic (e.g. *F*, *t*, *r*) with confidence intervals, effect sizes, degrees of freedom and *P* value noted<br>*Give P values as exact values whenever suitable.*
☒	☐	For Bayesian analysis, information on the choice of priors and Markov chain Monte Carlo settings
☒	☐	For hierarchical and complex designs, identification of the appropriate level for tests and full reporting of outcomes
☒	☐	Estimates of effect sizes (e.g. Cohen's *d*, Pearson's *r*), indicating how they were calculated

Our web collection on statistics for biologists contains articles on many of the points above.

Software and code

Policy information about availability of computer code

Data collection	The sample was collected using Tweepy (version 3.6.0), a library in Python that allows the use of the Twitter API to obtain tweets from Twitter that meet certain criteria. We wrote custom code to utilize Tweepy and extract variables from the tweets.
Data analysis	All statistical analysis was performed in SPSS version 24.

For manuscripts utilizing custom algorithms or software that are central to the research but not yet described in published literature, software must be made available to editors and reviewers. We strongly encourage code deposition in a community repository (e.g. GitHub). See the Nature Portfolio guidelines for submitting code & software for further information.

Data

Policy information about availability of data

All manuscripts must include a data availability statement. This statement should provide the following information, where applicable:

- Accession codes, unique identifiers, or web links for publicly available datasets
- A description of any restrictions on data availability
- For clinical datasets or third party data, please ensure that the statement adheres to our policy

The datasets generated and analysed during the current study are available from the corresponding author on reasonable request.

Field-specific reporting

Please select the one below that is the best fit for your research. If you are not sure, read the appropriate sections before making your selection.

☐ Life sciences ☒ Behavioural & social sciences ☐ Ecological, evolutionary & environmental sciences

For a reference copy of the document with all sections, see nature.com/documents/nr-reporting-summary-flat.pdf

Behavioural & social sciences study design

All studies must disclose on these points even when the disclosure is negative.

Study description	This is a proof of concept study, converting tweets into quantitative data to demonstrate how machine learning can identify stigma based on service user defined categories.
Research sample	The research sample was a set of tweets discussing schizophrenia with the posters of them originating from a wide range of countries, the sample is representative of tweets discussing schizophrenia at this time.
Sampling strategy	This sample was obtained through Tweepy, which gathered tweets containing at least one word from a list of keywords associated with Schizophrenia, through its own algorithm. We ran the code to obtain tweets in 5 different 48 hour periods. No sample size calculation was performed as we wanted a set large enough to encapsulate sufficient variance in tweets not to perform specific tests on. We could then draw a random sample from this set for each stage of the study, the size of each was based on how many tweets service users felt was feasible to rate collectively.
Data collection	Data was collected about the tweets using a mix of custom code and set algorithms, service users assigned the tweets a category of stigmatizing or not using an excel spreadsheet of all tweets, inputting the assigned category beside the tweet.
Timing	The tweets were collected in 5 different 48 hour periods between the 30th of January to the 11th of May: the 30th to the 31st of January, the 15th to the 16th of March, the 16th to the 17th of April, the 24th to the 25th of April and the 10th to the 11th of May.
Data exclusions	Tweets were removed from data collection if they were not sufficiently in English or lacked necessary context.
Non-participation	There were no research participants.
Randomization	No experimental groups were used within this study, so no assignment to condition, random or otherwise, took place.

Reporting for specific materials, systems and methods

We require information from authors about some types of materials, experimental systems and methods used in many studies. Here, indicate whether each material, system or method listed is relevant to your study. If you are not sure if a list item applies to your research, read the appropriate section before selecting a response.

Materials & experimental systems

n/a	Involved in the study	
☒	☐	Antibodies
☒	☐	Eukaryotic cell lines
☒	☐	Palaeontology and archaeology
☒	☐	Animals and other organisms
☒	☐	Human research participants
☒	☐	Clinical data
☒	☐	Dual use research of concern

Methods

n/a	Involved in the study	
☒	☐	ChIP-seq
☒	☐	Flow cytometry
☒	☐	MRI-based neuroimaging
